# A Treatise on Hare-Lip, and Its Treatment

**Published:** 1844-10

**Authors:** 


Art. X.—*A Treatise on Hare-Lip, and its Treatment.* By Dr. S. P. Hullihen, of Wheeling, Va. Baltimore: Woods & Crane. 1844. 8vo. pp. 12.

This is a sensible, well written pamphlet, the author of which seems to be master of his subject. We can readily believe that he treats successfully the very offensive deformity which he appears to have made the subject of special study. He is in favor of operating early, and offers strong reasons for performing the operation on the infant before dentition commences.

"The operation on infants, for the cure of hare-lip before the period of dentition has commenced, is more easily accomplished, presents more facilities for the complete removal of the deformity, and is less fraught with danger to the infant, and to the success of the operation, than at any other period.

"The infant, before dentition commences, has no fears of an operation, and therefore makes no resistance nor struggles, except those excited by the painful manipulations of the operator; and these being but momentary, and the child easily managed, the lip can be more satisfactorily prepared, and elegantly adjusted, than can possibly be accomplished on a child a few more months advanced in life. This circumstance, alone, is of much importance.

"The facilities for the more complete removal of the deformity of hare-lip before dentition commences, are very great and very important, where the deformity occurs in connecnection with a cleft in the alveolar and palatine arches. The bones of the face, at this period, being in a soft and cartilaginous state, can readily be brought into any desired position. The cleft in the alveolar arch can therefore be closed, its projections connected, its arch restored, which is as indispensbly necessary to the complete removal of the deformity, as a perfect adaptation of the lip. In addition to all this, nature makes a greater and more successful effort to restore all deficiences at this period of life than at any other.

"The operation before dentition commences, is likewise less fraught with danger to the infant and to the success of the operation than at any other period. Less fraught with danger to the infant, because the irritation consequent upon the operation can be rendered harmless, in all cases, by proper preparatory treatment; and because an infant is much less subject to convulsions before dentition, than after this process has commenced; neither is it liable to a host of other symptomatic diseases that so frequently accompany dentition, endangering and destroying life, independent of the consequences that might be added by the effects of an untimely operation. It is less dangerous to the success of an operation, because, at this time of life, an infant sleeps more than at any other, is less disposed to fret and cry, is less liable to disturb the lip and dressings with its hands, and is far more easily managed in every way that tends to the security and successful termination of the case.

"I am, therefore, decidedly in favor of early operations on infants, for the cure of hare-lip. I have operated on thirteen cases before dentition commenced, three infants of this number were only four weeks old; and I have yet to witness the first untoward event, or the slightest unfavorable indication resulting to an infant from the operation."

We shall quote the part of his pamphlet relating to the preparatory treatment, which consists in restoring the alveolar arch to its proper form, before the operation for hare-lip is undertaken. His description of it is as follows:

"A cleft of the alveolar and palatine arches, like that of the lip, is a congenital separation ot the parts, with but little if any loss of substance. Its connection, therefore, with hare-lip greatly increases the deformity of the whole countenance. The edges of the lip formed by the fissure are always carried apart as much further than is usual in simple cases of hare-lip, as the cleft may be wide in the alveolar arch. The nostril over the cleft is also stretched out and depressed; a projection of the alveolar process frequently occurs, and the face is always very perceptibly widened, all resulting from the cleft in the jaw, and all increasing or diminishing in deformity, in proportion as the cleft may vary in width.

"To unite the edges of hare-lip, where this complication of the deformity exists, is always more or less difficult, and sometimes even impossible, and when accomplished will not restore the form of the nostril, correct the projections of the alveolar process, nor relieve the unseemly width of the face, except in a very limited degree. But closing the cleft in the alveolar arch corrects, at once, all these irregularities, and at the same time approximates the edges of the lip so closely that they may be most admirably united, without the least danger to the infant or to the success of the operation. It is upon these grounds that the utility and importance of preparatory treatment is urged.

"The closure of the cleft in the alveolar arch may be effected in a variety of ways; but the most simple, and at the same time the most effectual, may be accomplished solely by the use of the adhesive strap, properly applied upon the cheeks.

"The cartilaginous state of the bones in early infancy requires but little force to bring them into any desired position. But in removing the deformity of a cleft alveolar arch, a force of a twofold nature is often required, both to bring the edges together and at the same time compress any projections of the alveolar process which may exist. In the proper application of the adhesive strap may be found this happy combination.

"The form of the strap which I have usually employed for this purpose is represented in the following cut.* It should be left as large at each end as the size of the cheek will permit, and slitted at different places, so that it may adhere smoothly and firmly. The part required to pass over the lip should be somewhat less than half an inch in width, the edges of this part being doubled over and fastened together, in order to give it the necessary strength and stiffness.

"The strap may be applied in the following manner: after being properly warmed, one end should be quickly and well adhered to the cheek of one side, then, pressing both cheeks forward, and passing the strap over the upper lip, close to the nose, it should be adhered in like manner to the cheek of the other side. By thus confining the cheeks forward, a force is obtained and exerted upon the jaw, sufficiently great to close in a few weeks the widest cleft in the alveolar arch, and at the same time to correct any projections of its process. The strap should be kept perfectly tense. It is therefore necessary to tighten it every day or two, which may be done by cutting a small portion out of the narrow part, and then sewing it together, without disturbing its adhesions to either cheek. In this way, the same strap will last for several days, and is so easily tightened that its management may be safely entrusted to the parents of the child. As the wearing of the strap never excoriates the parts, nor produces the least pain to the infant, however young, it is advisable to apply it as soon after birth as possible, as a cleft in the alveolar arch is more easily closed at this period than at any other; and as the strap is always of very great assistance to the infant in taking its food. In cases of simple hare-lip, without any cleft in the alveolar arch, the use of the strap will enable the child to nurse at the breast with but little if any difficulty."

---

* We omit the cut.

